# Low serum magnesium concentration is associated with the presence of viable hepatocellular carcinoma tissue in cirrhotic patients

**DOI:** 10.1038/s41598-021-94509-6

**Published:** 2021-07-26

**Authors:** Simona Parisse, Flaminia Ferri, Marzia Persichetti, Monica Mischitelli, Aurelio Abbatecola, Michele Di Martino, Quirino Lai, Sara Carnevale, Pierleone Lucatelli, Mario Bezzi, Massimo Rossi, Adriano De Santis, Alessandra Spagnoli, Stefano Ginanni Corradini

**Affiliations:** 1 Department of Translational and Precision Medicine, Sapienza University of Rome, Viale dell’Università 37, 00185 Rome, Italy; 2 Department of Radiological, Oncological and Anatomopathological Sciences, Sapienza University of Rome, Rome, Italy; 3 Hepato-Bilio-Pancreatic and Liver Transplant Unit, Department of Surgery, Sapienza University of Rome, Rome, Italy; 4 Department of Public Health and Infectious Diseases, Sapienza University of Rome, Rome, Italy

**Keywords:** Hepatocellular carcinoma, Hepatocellular carcinoma

## Abstract

This study aimed to ascertain, for the first time, whether serum magnesium (Mg) concentration is affected by the presence of hepatocellular carcinoma (HCC). We retrospectively enrolled consecutive cirrhotic patients with a diagnosis of HCC (n = 130) or without subsequent evidence of HCC during surveillance (n = 161). Serum levels of Mg were significantly (P < 0.001) lower in patients with HCC than in those without (median [interquartile range]: 1.80 [1.62–1.90] mg/dl vs. 1.90 [1.72–2.08] mg/dl). On multivariate logistic regression, low serum Mg was associated with the presence of HCC (OR 0.047, 95% CI 0.015–0.164; P < 0.0001), independently from factors that can influence magnesaemia and HCC development. In a subset of 94 patients with HCC, a linear mixed effects model adjusted for confounders showed that serum Mg at diagnosis of HCC was lower than before diagnosis of the tumor (β = 0.117, 95% CI 0.039–0.194, P = 0.0035) and compared to after locoregional treatment of HCC (β = 0.079, 95% CI 0.010–0.149, P = 0.0259), with two thirds of patients experiencing these changes of serum Mg over time. We hypothesize that most HCCs, like other cancers, may be avid for Mg and behave like a Mg trap, disturbing the body’s Mg balance and resulting in lowering of serum Mg levels.

## Introduction

Magnesium (Mg), as a co-factor for up to 600 enzymes, has a fundamental role in many physiological and biochemical functions including cell proliferation, DNA repair and energy metabolism^1–3^.

The available data indicate a double and opposite effect of the availability of Mg in the oncology field. In fact, on the one hand, a high content of Mg in the diet seems to be associated with a lower incidence of gastric, colon and breast cancers^4–6^. On the other hand, however, various data have shown how the availability of Mg by cancerous tissues could be involved in the development and/or growth of tumors^7–9^. In fact, some experimental studies have shown that Mg increases the proliferative capacity of tumors^7–9^. Furthermore, in vivo, breast cancer accumulates Mg causing a lowering of its concentration in serum in dogs and, in humans, the presence of head and neck cancers is associated with a reduction in serum Mg and magnesemia increases after radiotherapy of the tumor^10,11^. These data suggest that the neoplastic tissue would be avid for Mg to grow, causing a lowering of magnesemia, as also confirmed by the fact that in humans many other types of cancer are associated with reduced serum Mg levels^10–16^. This hypothesis is reinforced by the fact that an overexpression and/or activation of the Mg chanzyme channel, the transient receptor potential cation channel, subfamily M, member 7 (TRPM7), has been found in numerous cancers^17–24^. Based on these studies, therapeutic strategies with anti TRPM7 drugs have been designed in cancer patients^17,23,24^.

Primary liver cancers represent the 6th most frequent malignancy and the 4th leading cause of cancer death worldwide^25,26^. A negative association with dietary intake of Mg in humans has also been demonstrated for primary liver cancers, but there are no data on serum Mg levels in these patients and, therefore, it is not known whether liver cancer causes a reduction in serum Mg^27–29^. Hepatocellular carcinoma (HCC) accounts for approximately 75–85% of primary liver cancers and is more common in patients with liver cirrhosis of different etiologies^25,26^. Patients with liver cirrhosis have low serum and intracellular Mg levels^29^.

Thus, in the present retrospective study we wanted to compare the Mg levels in the serum of cirrhotic patients with or without HCC, adjusting for several confounding factors that could influence magnesemia. Furthermore, in patients with HCC, we wanted to verify how the serum concentration of Mg varied over time, comparing the values before the diagnosis of HCC, at the time of diagnosis of the tumor and after its treatment.

## Methods

### Study population

The study analyzed 637 consecutive cirrhotic patients, evaluated at the Liver Transplant outpatient Service of Gastroenterology Division at Sapienza University of Rome (Italy) from February 1999 to January 2020. Only patients with an available Mg concentration at the beginning of cirrhosis follow-up and/or at HCC diagnosis and a minimum follow-up period of 6 months were enrolled in the study (n = 317). Twenty-six patients taking Mg supplements at the time of serum Mg determination were excluded. In the case–control study were included 291 patients, divided in two groups according to the absence or presence of HCC. The group without HCC comprised 161 cirrhotic patients under a every 6-month ultrasound HCC surveillance program but without evidence of HCC until the end of follow-up. The group with HCC comprised 130 cirrhotic patients with a diagnosis of HCC, according to the most up-date EASL guidelines available at the time of enrollment^30–32^. The Milan criteria for liver transplantation were used to classify patients with HCC based on the size and number of nodules (single tumor less than or equal to 5 cm, or up to 3 tumors, each less than or equal to 3 cm)^33^.

One hundred twenty-two of the enrolled patients were transplanted during the study period, including 67 without and 55 with HCC. Demographic and clinical data, as well as potential factors that may influence serum Mg^34^ were retrieved for each patient at the time of blood sampling for Mg determination. Hypomagnesaemia was defined as a serum Mg concentration below 1.7 mg/dl.

Information about the Mg and calcium dietary intake were available in 91 patients (52 cirrhotic without HCC and 39 cirrhotic with HCC) and were collected using a 3-day food record. The records were analyzed through the Food Composition Database for Epidemiological Studies in Italy (BDA)^35^.

We then wanted to study the temporal changes in serum Mg concentration of patients with HCC, comparing serum Mg before HCC diagnosis, at the time of tumor diagnosis and after locoregional therapy. For this purpose we therefore considered 41 cirrhotic patients with HCC who, in addition to the serum Mg concentration measured at the time of diagnosis of the tumor, also had a serum Mg measurement available at least 6 months before diagnosis. In 15 of these patients, measurement of serum Mg was also available at least 6 months after the start of locoregional therapy. We also considered an additional 38 patients in the study diagnosed with HCC for whom, although measurement of serum Mg was not available before the diagnosis of HCC, we had it at the time of diagnosis and at least 6 months after the start of locoregional therapy.

### Statistical methods

Continuous variables were expressed as median and interquartile range (IQR) or mean and standard deviation according to their distribution. Categorical data were recorded as frequencies and percentages. The differences between patients with and without HCC were evaluated by the Mann–Whitney U test, the T test or the chi-square test, as appropriate.

Multivariable binary logistic regression models were used to estimate the association of demographic and clinical variables with the presence of HCC. All variables with a P-value < 0.05 on univariate analyzes were entered as covariates and the sex and etiology of cirrhosis, although not significant on univariate analyzes, were considered as adjustment covariates. Model selection was performed by stepwise procedure based on the Akaike Information Criterion.

In HCC patients, a mixed effects model was applied to estimate the effect of demographic and clinical variables on serum Mg concentration at different time points. This model included the categorical time point (before HCC diagnosis, at HCC diagnosis, after treatment), diabetes, MELD score, administration of non-absorbable disaccharides, and a subject-specific random intercept to take into account dependence between repeated measurements on the same subject. The administration of furosemide and potassium-sparing diuretics were included as adjustment variables. Correlations between temporal changes in serum Mg concentration and the total tumor volume of HCC were estimated using Spearman’s rho coefficient. The tumor volume of each HCC nodule was calculated with the formula: tumor volume (cm^3^) = 4/3 × 3.14 × (maximum radius of the tumor nodule in cm)^3^. The total tumor volume was calculated as the sum of the tumor volumes of every nodule^36^. The diagnostic performance of the serum Mg concentration at diagnosis was assessed by the area under the curve (AUC) plotting receiver operating characteristic (ROC) curve that was designed to differentiate between the patients with and without HCC. Optimal cut-off value was chosen to maximize the sum of sensitivity and specificity.

All tests were two-tailed, and a P-value < 0.05 was considered as statistically significant. Analyses were performed using R version 4.1.0 (The R Project for Statistical Computing)^37^.

### Ethics approval

This observational study was approved by the Ethics Committee of the “Sapienza University of Rome-Policlinico Umberto I” as a spin-off of another ongoing study on the “Effects of soluble products and extravesicular vesicles derived from the intestinal microbiota on the tumorigenesis of hepatocellular carcinoma” (Ref. N. 3420) and was conducted in accordance with the principles of good clinical practice and the Italian laws. The study was conducted following the guidelines outlined in the Declaration of Helsinki.

### Consent to participate

Informed consent has been collected from all participants.

### Consent for publication

All authors agreed to publish this manuscript.

## Results

### The serum Mg concentration is lower in cirrhotic patients with HCC than in those without liver cancer

To evaluate the absence of HCC, all cirrhotic patients without HCC were followed within an HCC surveillance program with ultrasound every 6 months for a minimum of 180 days and a median of 547 days (95% CI 256–913 days). Among the HCC patients none had metastases or vascular invasion and 106 (81.5%) were within the Milan criteria for liver transplantation. The demographic and clinical characteristics of the study participants are shown in Table 1, divided into two groups based on the presence or absence of HCC. The characteristics refer to the time of blood sampling to determine the concentration of Mg, corresponding to the first visit for patients without HCC and to the time of diagnosis of HCC for patients with cancer. In univariate analysis, patients with HCC were significantly older than cirrhotic patients without HCC. Patients with HCC had a higher BMI, with median values of both groups in the overweight range. The two groups had a different distribution of the etiologies of cirrhosis, with a smaller percentage of subjects with infrequent etiologies, such as Wilson’s disease, primary biliary cholangitis, primary sclerosing cholangitis, in the HCC group.

**Table 1:** Demographic and clinical characteristics according to the presence or absence of HCC in cirrhotic patients.

	Cirrhosis without HCC n = 161	Cirrhosis with HCC n = 130	P value
Age (years)	55.00 (49.10–60.04)	58.10 (51.67–62.80)	**0.001**
Gender M (%)	127 (78.9)	110 (84.6)	0.272
BMI (kg/m^2^)	25.34 (23.52–28.43)	26.67 (23.88—30.03)	**0.027**
Diabetes, yes, n (%)	41 (25.5)	38 (29.2)	0.473
Etiology of liver cirrhosis, n (%)			**0.033**
HCV	36 (22.4)	39 (30.0)	
HBV	10 (6.2)	9 (6.9)	
Alcohol	58 (36.0)	31 (23.8)	
Virus + alcohol	24 (14.9)	31 (23.8)	
Post-NASH	15 (9.3)	14 (10.8)	
Others (cholestatic, Wilson etc.)	18 (11.2)	6 (4.6)	
Ascites, yes (%)	97 (60.2)	50 (38.5)	** < 0.001**
Furosemide, yes (%)	105 (65.2)	61 (46.9)	**0.003**
Potassium-sparing diuretics, yes (%)	117 (72.7)	74 (56.9)	**0.007**
Nonabsorbable disaccharides, yes (%)	76 (47.2)	51 (39.2)	0.213
Levothyroxine, yes (%)	8 (5.0)	7 (5.4)	0.873
Proton pump inhibitors, yes (%)	92 (57.1)	72 (55.4)	0.856
MELD score	15.00 (12.00–19.00)	14.00 (11.00–17.00)	** < 0.001**
Serum Mg (mg/dl)	1.90 (1.72–2.08)	1.80 (1.62–1.90)	** < 0.001**
Hypomagnesemia, yes (%)	24 (14.9)	37 (28.5)	**0.007**
Serum Ca (mg/dl)	8.69 (± 0.61)	8.74 (± 0.67)	0.587
Mg in diet, mg*	93.20 (75.06–119.97)	93.03 (71.87–129.30)	0.990
Ca in diet, mg*	699.56 (± 258.49)	699.68 (± 274.60)	0.998

Continuous variables are shown as median and interquartile range or mean ± SD, according to the absence or presence of normal distribution. Categorical variables are shown as numbers and percentages. The characteristics refer to the time of blood sampling to determine the concentration of Mg, corresponding to the first visit for patients without HCC and to the time of diagnosis of HCC for patients with cancer.

*BMI* body mass index, *Ca* calcium, *HBV* hepatitis B virus, *HCC* hepatocellular carcinoma, *HCV* hepatitis C virus, *MELD* model for end-stage liver disease, *Mg* magnesium, *NASH* non-alcoholic steatohepatistis.

*Available only in 52 cirrhotic without HCC and 39 cirrhotic with HCC. p-values numbers marked in bold indicate numbers that are significant at the p < 0.05 level

Cirrhotic patients without HCC had a more advanced stage of cirrhosis than cirrhotic patients with HCC as evidenced by a higher MELD score, a greater proportion of subjects with ascites, and were more frequently treated with furosemide and potassium-sparing diuretics. No difference between the groups was present with regard to treatment with other drugs potentially capable of affecting serum Mg levels, such as proton pump inhibitors, levothyroxine and non-absorbable disaccharides. Despite similar dietary intakes of Mg in the two groups, cirrhotic patients with HCC had a significantly lower serum concentration of Mg than cirrhotic patients without HCC. Hypomagnesaemia was more frequent in the HCC group than in the non-HCC group. Serum levels and dietary calcium intake did not differ between the two groups (Table 1).

In a multivariable stepwise logistic regression model, adjusted for gender and for the etiology of cirrhosis, the variables that remained significantly associated with the presence of HCC were BMI, age, absence of potassium sparing diuretic administration, low MELD score and low serum concentration of Mg. The serum concentration of Mg was associated with very low odds of HCC (OR 0.047, 95% C.I. 0.015–0.164; P < 0.0001) (Table 2). When, instead of the serum Mg concentration values, the presence of hypomagnesemia was considered in the model, hypomagnesemia was also associated with the presence of HCC (OR 3.443, 95% C.I. 1.750–6.995; P < 0.0001). Finally, although the dietary intake of Mg was available only in a subgroup of patients, adjusting the logistic regression model also for the dietary intake of Mg, the concentration of Mg in serum was associated with even lower odds of HCC (OR 0.005, 95% CI 0.000–0.050; P < 0.001).

**Table 2:** Factors associated with the presence of HCC at stepwise multivariate binary logistic regression analysis.

	OR	95% CI	P value
Age (years)	1.061	1.025–1.102	**0.00127**
BMI (kg/m^2^)	1.094	1.028–1.167	**0.00520**
Potassium-sparing diuretics, yes vs no	0.529	0.289–0.963	**0.03777**
MELD score	0.852	0.793–0.909	**< 0.0001**
Serum Mg (mg/dl)	0.047	0.014–0.146	**< 0.0001**

Model adjusted for cirrhosis etiology and gender.

*BMI* body mass index, *HCC* hepatocellular carcinoma, *MELD* model for end-stage liver disease, *Mg* magnesium. p-values numbers marked in bold indicate numbers that are significant at the p < 0.05 level

The sensitivity and specificity of serum Mg concentration for the presence of HCC were, with a cutoff value of 1.997 mg/dl, 0.8538 (95% CI 0.7729–0.9154) and 0.413 (95% CI 0.3446–0.4848), respectively.

### The serum concentration of Mg decreases at the diagnosis of HCC and increases after locoregional therapy of the tumor

**Figure 1:**
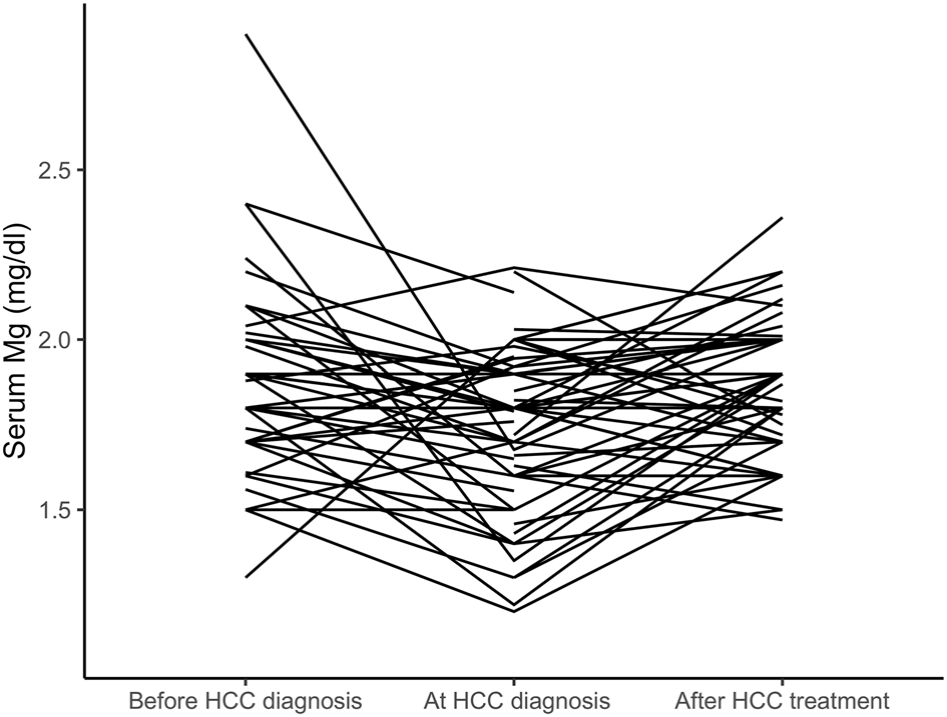
Spaghetti plot of serum Mg concentration in patients with HCC before tumor diagnosis, at the time of diagnosis and after locoregional treatment.

**Figure 2:**
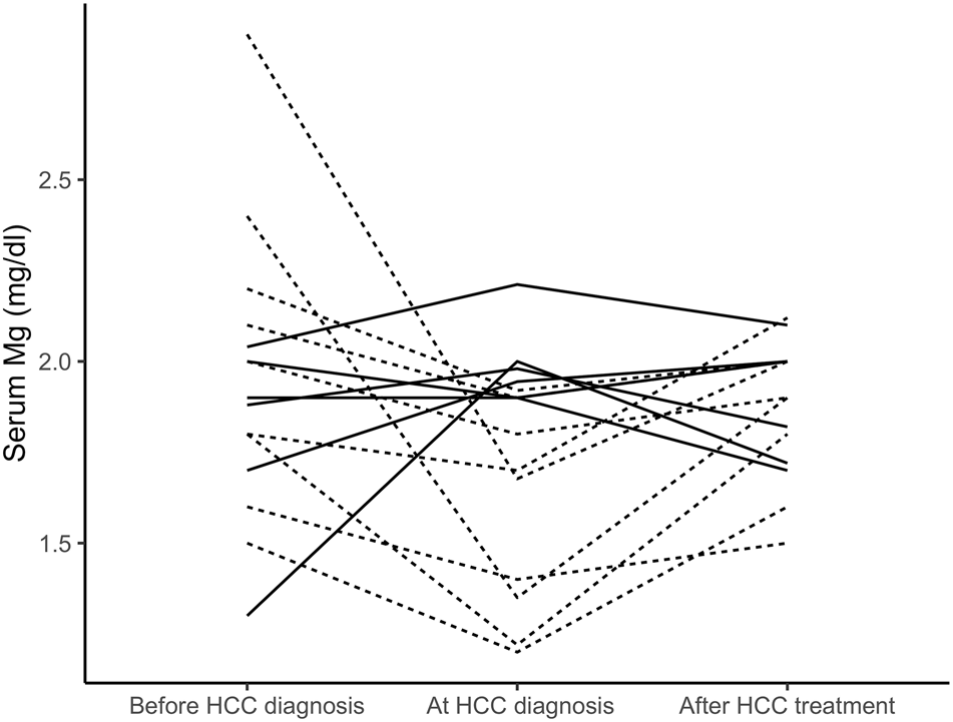
Spaghetti plot for the 15 patients in whom serum Mg concentrations were available at all three time points (i.e. before HCC diagnosis, at tumor diagnosis and after locoregional treatment). The dotted lines represent patients in whom the serum Mg concentration decreased at the diagnosis of HCC compared to before and then increased subsequently after locoregional treatment. Other patients who did not show such temporal changes in serum Mg are represented by the solid lines.

In 41 cirrhotic patients with HCC, in addition to the serum Mg concentration measured at the time of diagnosis of the tumor, serum Mg measured at least 6 months before the time of diagnosis of the tumor was also available. In 15 of these patients and in a further 38 of the patients in the study diagnosed with HCC, serum Mg measured at least 6 months after the start of locoregional therapy was also available. The mean time elapsed between the measurement of serum Mg concentration before the diagnosis of HCC and the diagnosis of the tumor was 508 ± 305 days. The mean time elapsed between the date of the first locoregional HCC treatment and the day of serum Mg measurement after treatment was 401 ± 288 days. The first locoregional treatment was surgery (n = 6), radio-frequency thermal ablation (n = 16), transarterial chemoembolization (n = 31) and transarterial radioembolization (n = 1). Individual serum Mg concentration data before HCC diagnosis, at tumor diagnosis and after locoregional therapy are shown as spaghetti plot in Fig. 1. The linear mixed-effects model, adjusted for the administration of furosemide and potassium-sparing diuretics (Table 3), showed that the serum Mg concentration at the diagnosis of HCC was lower than before the diagnosis of HCC (β = 0.117, 95% CI 0.039–0.194, P = 0.0035) and compared to after the treatment of HCC (β = 0.079, 95% CI 0.010–0.149, P = 0.0259). When we analyzed all 41 patients for whom we had data on the change in serum Mg concentration at the time of HCC diagnosis compared to before tumor development, 27 patients (66%) showed a reduction in magnesemia at diagnosis. Furthermore, the change in magnesemia was not related to the total tumor size. When we analyzed only 34 patients with HCC within the Milan criteria for liver transplantation, 23 patients (68%) showed a reduction in magnesemia at tumor diagnosis. In these patients we found an inverse correlation between temporal changes in serum Mg levels before and at the time of tumor diagnosis (delta serum Mg at diagnosis = Mg at diagnosis − Mg before diagnosis) and the total tumor volume of HCC (r =  − 0.372; P = 0.031) (Supplementary Fig. S1). When we analyzed all 53 patients for whom we had data on the change in serum Mg concentration after locoregional HCC treatment compared to the time of diagnosis, 33 patients (63%) showed an increase in magnesemia after therapy. When we analyzed only 39 patients with HCC within the Milan criteria for liver transplantation, 26 patients (67%) showed an increase in magnesemia after therapy. Furthermore, the change in magnesemia was not related to the total tumor volume before treatment (data not shown) and did not differ in patients with complete response and no HCC recurrence compared to those with partial response to treatment and/or HCC recurrence (0.71 ± 0.46 mg/dl versus 0.46 ± 0.51 mg/dl, respectively, P = 0.084). Figure 2 shows the spaghetti plot for the 15 patients in whom serum Mg concentrations were available at all three time points, i.e. before HCC diagnosis, at tumor diagnosis and after locoregional treatment. The patients in whom serum Mg was reduced at the diagnosis of HCC compared to before were those in whom serum Mg increased after locoregional treatment. Indeed, there was a strong negative correlation between the change in serum Mg at diagnosis of HCC (serum delta Mg at diagnosis = serum Mg at diagnosis − serum Mg before diagnosis) and the change in serum Mg after locoregional treatments (serum delta Mg after treatment = serum Mg after treatment − serum Mg at diagnosis) (r =  − 0.746; P = 0.001).

**Table 3:** Linear mixed-effect model of serum Mg concentration at different time points.

Variable	β Coefficient	95% CI	P-value
Before HCC diagnosis vs at HCC diagnosis	0.117	0.039 to 0.194	**0.0035**
After locoregional therapy vs at HCC diagnosis	0.079	0.010 to 0.149	**0.0259**
Diabete yes vs no	− 0.109	− 0.186 to − 0.033	**0.0055**
MELD	− 0.009	− 0.018 to 0.0001	0.0536
Nonabsorbable disaccharides, yes vs no	− 0.051	− 0.127 to 0.024	0.1805

Linear mixed effect model adjusted for furosemide and potassium-sparing diuretic administration.

*HCC* hepatocellular carcinoma, *MELD* model for end-stage liver disease. p-values numbers marked in bold indicate numbers that are significant at the p < 0.05 level

## Discussion

In this study we demonstrated, for the first time, that cirrhotic patients with HCC have a lower serum concentration of Mg than those without HCC, regardless of confounding factors, including dietary intake of Mg and drugs that can alter magnesaemia^34^. The association of low serum Mg concentration with HCC is consistent with the hypothesis that HCC, like other tumors, may be avid for Mg to support cell proliferation and may act as a Mg trap, disturbing the balance of the body’s Mg and resulting in lower serum Mg concentrations^10–16^.

This hypothesis is further confirmed by our current results regarding temporal changes in serum Mg concentration in patients who develop HCC and then undergo locoregional treatments. First, we found that the serum concentration of Mg decreases at the time of diagnosis of HCC compared to the values present before the development of the tumor and then, similarly to what has been demonstrated after radiotherapy in patients with head and neck cancer^11^, it increases after locoregional treatments compared to the values present at the diagnosis of the tumor. Second, patients with a more pronounced decrease in serum Mg concentration at the time of HCC diagnosis compared to that before tumor development are those with a more pronounced subsequent increase in serum Mg after locoregional treatment. This suggests that some HCCs have a high avidity for Mg which results in the reduction of magnesemia. Once these tumors are then necrotized or eliminated by therapy, they no longer remove Mg from the blood and magnesemia increases. Cellular Mg homeostasis is guaranteed mostly by TRPM7. TRPM7 is a ubiquitous chanzyme with an ion channel permeable to divalent cations (Mg^2+^, Ca^2+^, Zn^2+^) and an alpha-kinase domain involved in intracellular signalling (receptor tyrosine kinase-mediated pathways and growth factors mostly)^18^. Studies have pointed out that TRPM7 overexpression and/or activation is involved in growth and proliferation of several cancers (glioma, gastric, oesophageal, lung, breast, ovary, bladder, prostate, retinoblastoma, colon, pancreas, head and neck, nasopharyngeal)^17–24^ and therapeutic strategies with anti TRPM7 drugs have been designed in cancer patients^17,23,24^. Our current data are a stimulus to study the expression of TRPM7 in HCC cells and to verify whether, as found in glioma cells, their Mg content is increased^38^. Furthermore, it will be interesting to verify the expression of TRPM7 and the Mg content in different HCCs classified according to their molecular activation pathways involved in tumor formation/progression^39^. In fact, approximately 30% of the HCC patients in our study did not have the changes in serum Mg at the time of cancer diagnosis and after treatment consistent with neoplastic tissue avidity for Mg. In this subgroup of patients, HCC may have non-Mg dependent molecular activation pathways and/or not have increased TRPM7 expression/activation. In this regard it is known that two of the most frequent molecular activation pathways of HCC, telomere maintenance and cell cycle control mediated by the p53 tumor cell antigen are known to be affected by Mg, but the other activation pathways may be insensitive to the cation^39–41^. This heterogeneity of HCC in accumulating and using Mg to grow could also partly explain why previous studies on other tumors have shown differences in serum Mg concentrations between study and control groups greater than those which we found in our study^11,12,14,15^. It should be noted that in our study we analyzed patients with non-metastatic HCC, mostly of limited size within the transplant criteria and found differences similar to those reported for patients with operable thyroid cancer^13^. In contrast, studies that showed greater differences in magnesaemia between patients with cancer and controls were performed in patients with head and neck or breast cancers with a high rate of metastases and advanced TNM staging^11,12,14^. Thus, the effect on tumor-induced reduction in serum magnesium appears to depend on the amount of neoplastic tissue, as verified in a study where the magnitude of reduction in magnesium in patients with lung, breast, pharyngeal and ovarian cancer correlated with the TNM stage^12^. In support of this hypothesis, in our patients with HCC within the Milan criteria for liver transplantation we found an inverse correlation between the reduction in magnesemia associated with the diagnosis of the tumor and the total tumor volume. However, when we also included larger tumors outside the transplantation criteria, the correlation does not remain, because very large tumors are associated with a lower than expected reduction effect on magnesium levels. The explanation for this particular behavior of larger tumors could be that, as already demonstrated, large HCCs are associated with severe spontaneous necrosis of the lesions and therefore have fewer viable cells capable of internalizing Mg than those expected based on the size of the lesions^42^. Regarding the change in magnesemia after locoregional treatment, we did not find a relationship either with the total tumor volume before treatment or with the residual tumor tissue after therapy. However, there was a trend for a greater increase in magnesemia in complete response and non-recurring tumors. This data could be explained by the too small sample size, considering also the aforementioned fact that about 30% of HCCs do not behave as a trap for Mg.

The data from our study indirectly demonstrating HCC avidity for Mg may appear to contradict the recently described association between primary liver cancer and low Mg in the diet^27,28^. This apparent contradiction has already been demonstrated for breast cancer and, although with not always consistent results regarding the protective role of mg in the diet, for colon cancer^4,6,12,14,16^. The contradiction could be explained on the one hand by a beneficial effect of Mg in the diet to prevent cancer but, on the other, by a favoring effect of Mg on the growth of cancer cells. Indeed, dietary Mg could protect against the development of HCC by reducing genomic instability and chronic inflammation in the cirrhotic liver, secondary to the Mg depletion that is known to be present in cirrhosis even in the absence of HCC. However, once HCC develops, cancer cells may become avid for Mg and use it to grow^29,43^.

At multivariate analysis, we found that a lower frequency of administration of potassium-sparing diuretics was also associated with HCC. This is in line with our hypothesis that most HCCs are avid for Mg. Indeed, eplerelone, an anti-aldosteronic drug, in addition to showing an antitumor action on HCC in mice by reducing angiogenesis, has been shown to antagonize the TRPM7-mediated increase in intracellular Mg induced by aldosterone, in embryonic human kidney cells^44,45^.

At multivariate analysis, we found that also older age and higher BMI were significantly associated with HCC, in line with previous reports^46,47^. Not surprisingly, a low MELD score was also associated with HCC and, in our opinion, this finding can be justified as population selection bias. Indeed, our entire cohort was enrolled in the Liver Transplant outpatient Service of Gastroenterology Division at Sapienza University of Rome (Italy), which by its own nature implies a selection bias: cirrhotic patients have a decompensated liver disease to be referred to our Transplant Center, whereas patients with HCC, that can be considered for transplantation, comprise also patients with compensated liver cirrhosis^48^. In addition, cirrhotic patients without HCC with poor liver function died of liver failure without developing cancer in the follow-up period.

Our study had some limitations. First, it is retrospective and monocentric and, second, the serum Mg concentrations that allowed the study of Mg temporal variations were only available in subgroups of patients.

In conclusion, we demonstrate for the first time that cirrhotic patients with HCC have reduced serum Mg concentrations compared to cirrhotic without HCC and that, in most HCCs, serum Mg levels decrease upon diagnosis of the tumor and increase after locoregional treatment. Further studies will be needed to identify the characteristics of HCC patients whose tumor is avid for Mg to grow and, from a precision medicine perspective, pave the way for new therapeutic strategies, similar to what has been proposed for the treatment of other solid tumors^18,23,24^.

## Supplementary Information

Supplementary Information.

## Data Availability

The data in this study are available from the corresponding authors on reasonable request.
