# Supplementary material for: Low serum magnesium concentration is associated with the presence of viable hepatocellular carcinoma tissue in cirrhotic patients
Source: Sci Rep. 2021 Jul 26;11:15184. doi: 10.1038/s41598-021-94509-6 (PMC8313704; doi:10.1038/s41598-021-94509-6)
Supplement: Supplementary file 1 — Supplementary Information. [file 41598_2021_94509_MOESM1_ESM.pdf]

Low serum magnesium concentration is associated with the presence of viable hepatocellular carcinoma tissue in cirrhotic patients.

Simona Parisse, Flaminia Ferri, Marzia Persichetti, Monica Mischitelli, Aurelio Abbatecola, Michele Di Martino, Quirino Lai, Sara Carnevale, Pierleone Lucatelli, Mario Bezzi, Massimo Rossi, Adriano De Santis, Alessandra Spagnoli, Stefano Ginanni Corradini.

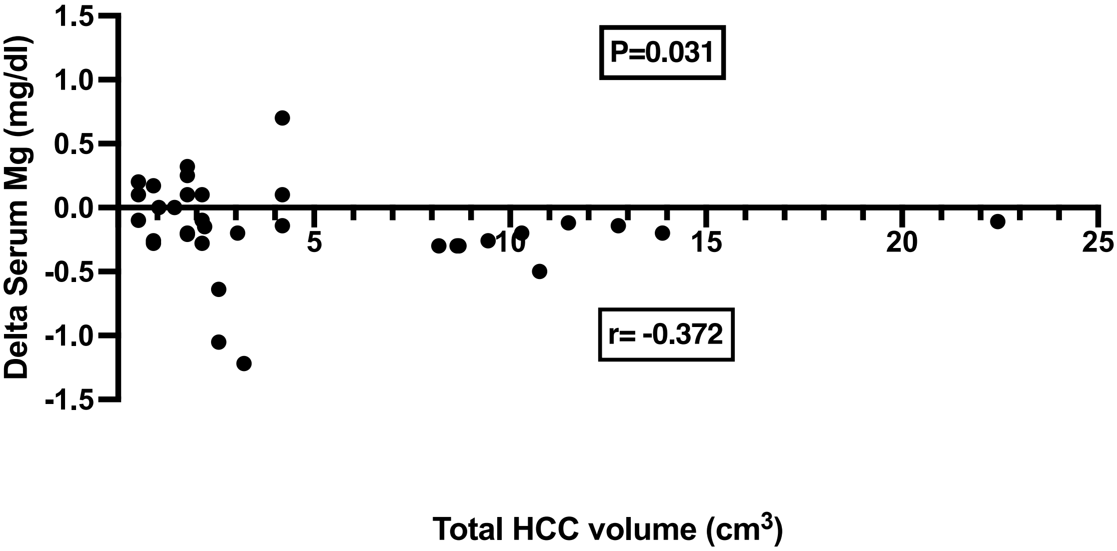

Supplementary Figure 1. Correlation between total HCC volume (the sum of the volumes of all nodules in each patient) with variations of serum Mg concentration at HCC diagnosis compared to that at least six months before (delta serum Mg = serum Mg at diagnosis – serum Mg before diagnosis)
